# Wall-Eyed Bilateral Internuclear Ophthalmoplegia With Concomitant Facial Diplegia: A Case Report and Review of the Literature

**DOI:** 10.7759/cureus.114810

**Published:** 2026-08-19

**Authors:** Imane Benderaz, Wadii Bnouhanna, Najoua Maarad, Noura Boukricha, Mounia Rahmani, Maria Benabdeljlil, Saadia Aïdi

**Affiliations:** 1 Department of Neurology A and Neuropsychology, Faculty of Medicine and Pharmacy, Specialties Hospital, Avicenne University Hospital, Mohammed V University, Rabat, MAR

**Keywords:** brain mri, facial diplegia, internuclear ophthalmoplegia, ischemic stroke, medial longitudinal fasciculus, one-and-a-half syndrome, paramedian pontine reticular formation, pontine tegmental infarction, webino syndrome

## Abstract

Wall-eyed bilateral internuclear ophthalmoplegia (WEBINO) syndrome is a rare neuro-ophthalmological condition. Its association with facial diplegia is even more uncommon, making this combination one of the rarest brainstem syndromes, with only a few cases reported in the literature. We report the case of a 62-year-old woman with multiple cardiovascular risk factors and a history of stroke attributed to small vessel disease. She presented with the sudden onset of diplopia and bilateral facial palsy. Neurological examination revealed WEBINO syndrome associated with facial diplegia. Brain magnetic resonance imaging demonstrated two bilateral acute ischemic lesions within the dorsal pontine tegmentum. Magnetic resonance angiography revealed atherosclerosis of the basilar artery and the right vertebral artery. Cardiac investigations, including an electrocardiogram and transthoracic echocardiography, were normal. Laboratory investigations were within normal limits apart from an elevated HbA1c.

The presence of two bilateral pontine tegmental infarcts, rather than the single midline lesion reported in previous cases, suggests possible involvement of two paramedian perforating arteries. We hypothesize that this pattern may reflect a vascular variant in which the paramedian perforating arteries share a common origin from the basilar artery. This case may help clinicians recognize this rare pontine tegmental syndrome and highlight a distinct radiological pattern in WEBINO syndrome with facial diplegia.

## Introduction

Wall-eyed bilateral internuclear ophthalmoplegia (WEBINO) syndrome is a rare neuro-ophthalmological syndrome characterized by bilateral adduction deficits, bilateral abducting nystagmus, and exotropia in primary gaze. It results from bilateral lesions of the medial longitudinal fasciculus (MLF), a brainstem pathway that coordinates horizontal conjugate eye movements by connecting the abducens nucleus with the contralateral oculomotor nucleus [1,2]. The MLF courses through the dorsal pontine tegmentum in close anatomical proximity to other cranial nerve nuclei and brainstem structures [1,2]. Therefore, the clinical presentation may vary according to the location of the lesion and the involvement of adjacent structures. A lesion extending beyond the MLF and involving the facial nerve nucleus or fascicles can consequently produce associated facial diplegia [3].

WEBINO syndrome with facial diplegia is extremely uncommon; to our knowledge, only three cases have previously been reported in the literature [4-6]. We report this case to highlight this rare syndrome and discuss the imaging findings and the possible underlying mechanism.

## Case presentation

A 62-year-old woman with a medical history of poorly controlled hypertension for several years, type 2 diabetes mellitus, and a lacunar stroke three years ago attributed to hypertensive small vessel disease, leaving residual right hemiparesis; her modified Rankin Scale (mRS) score was 3 prior to the current presentation [7]. In March 2026, she presented to the emergency department with sudden-onset binocular horizontal diplopia associated with exotropia observed by her family, with symptom onset 24 hours prior to arrival. The medications she used were amlodipine 10 mg, losartan 100 mg, and acetylsalicylic acid 160 mg.

**Figure 1:**
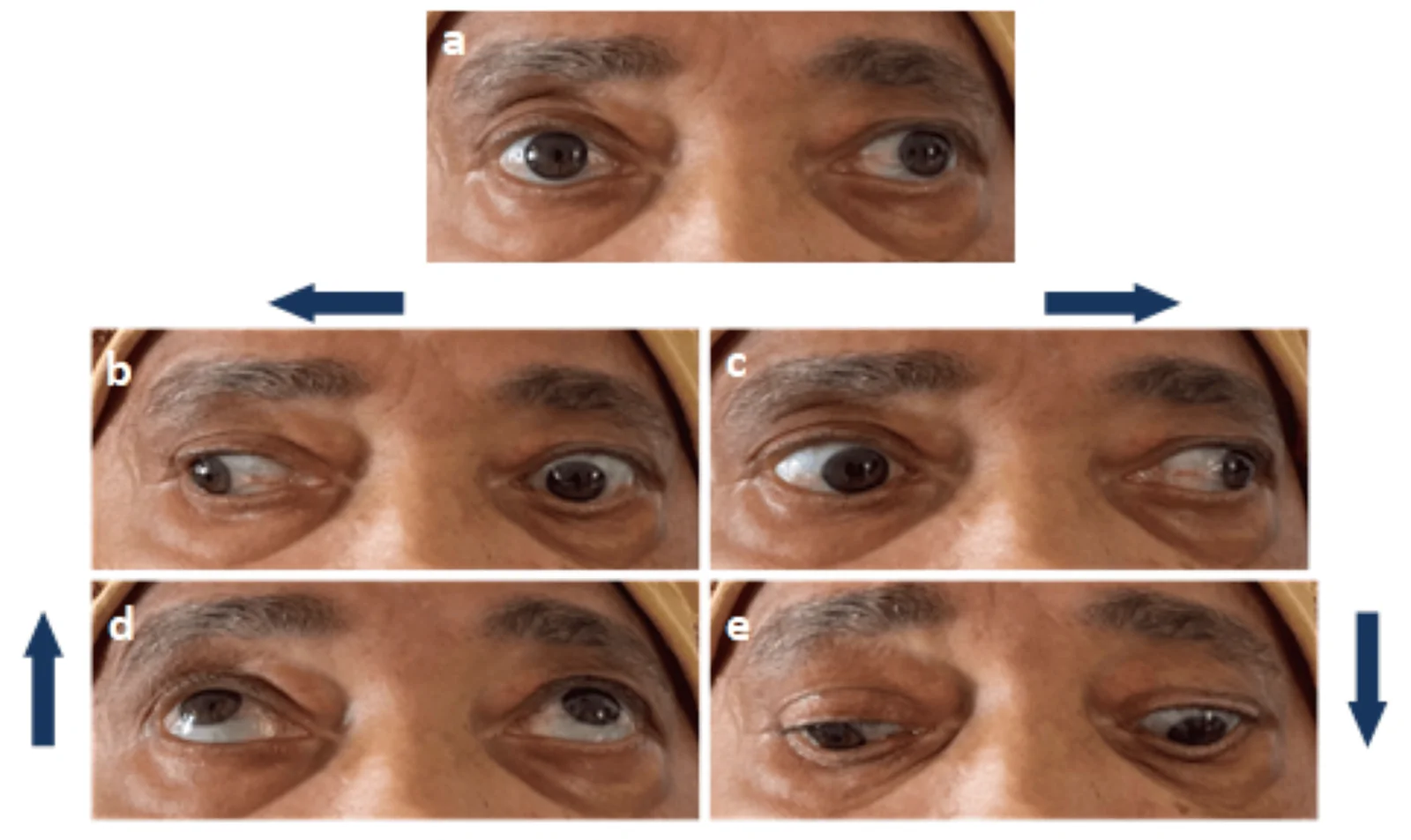
Ocular motility testing. (a) Primary gaze position showing bilateral exotropia, more pronounced in the right eye. (b, c) Horizontal gaze with fixation on the right and left eyes, respectively, demonstrating bilateral limitation of adduction, consistent with bilateral internuclear ophthalmoplegia. (d, e) Upward and downward gaze, showing preserved vertical eye movements.

**Figure 2:**
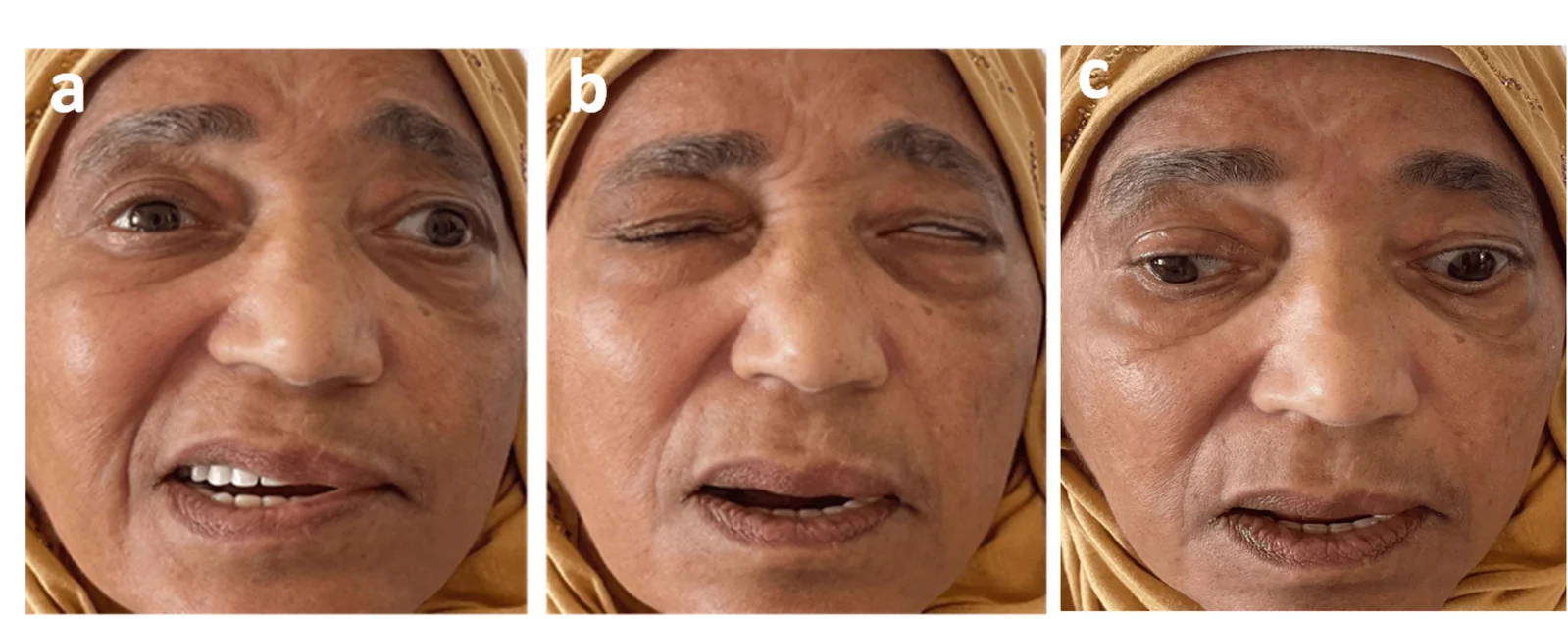
Cranial nerve examination showing bilateral facial diplegia predominantly affecting the left side. (a) Bilateral weakness of the lower facial muscles when smiling, predominantly on the left side. (b) Attempted eyelid closure showing Bell's phenomenon on the left and eyelash sign on the right side. (c) Bilateral weakness in forehead wrinkling. Written consent was obtained for publication of the photograph.

On admission, her blood pressure was 120/87 mmHg and capillary blood glucose was 1.20 g/L (<1.4). Neurological examination revealed bilateral exotropia, more pronounced in the right eye, in the primary gaze, bilateral limitation of adduction with preserved abduction, and abducting nystagmus of both eyes on horizontal gaze (Figure 1). Vertical gaze and convergence were intact. The examination also revealed peripheral facial diplegia, predominantly affecting the left side (Figure 2), without involvement of other cranial nerves. Her residual right hemiparesis was unchanged, with no evidence of new limb weakness. Cardiovascular and systemic examinations were unremarkable.

**Figure 3:**
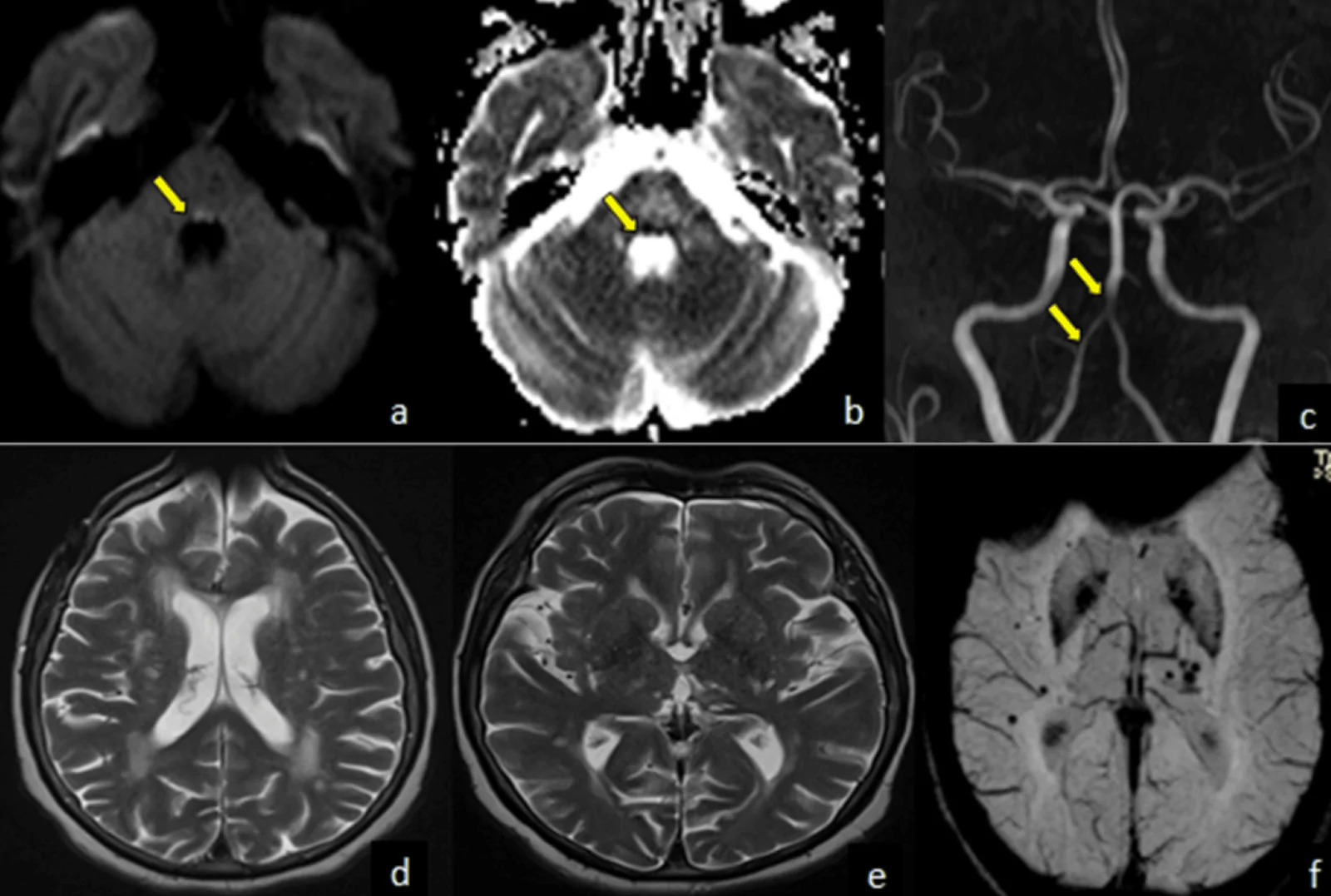
Brain MRI. Diffusion-weighted imaging (DWI) of the brainstem showing two adjacent hyperintense foci within the pontine tegmentum (arrow) (a), with corresponding restricted diffusion on the apparent diffusion coefficient (ADC) (arrow) (b), suggestive of acute bilateral dorsal pontine tegmental infarction. Atheroma in the proximal third of the basilar artery and right vertebral artery on MR angiography (arrows) (c). Vascular leukoencephalopathy (d) and dilated Virchow-Robin spaces (e) on T2-weighted images. Cerebral microbleeds on SWI sequence (f), related to cerebral small vessel disease. SWI: Susceptibility-weighted sequence

**Figure 4:**
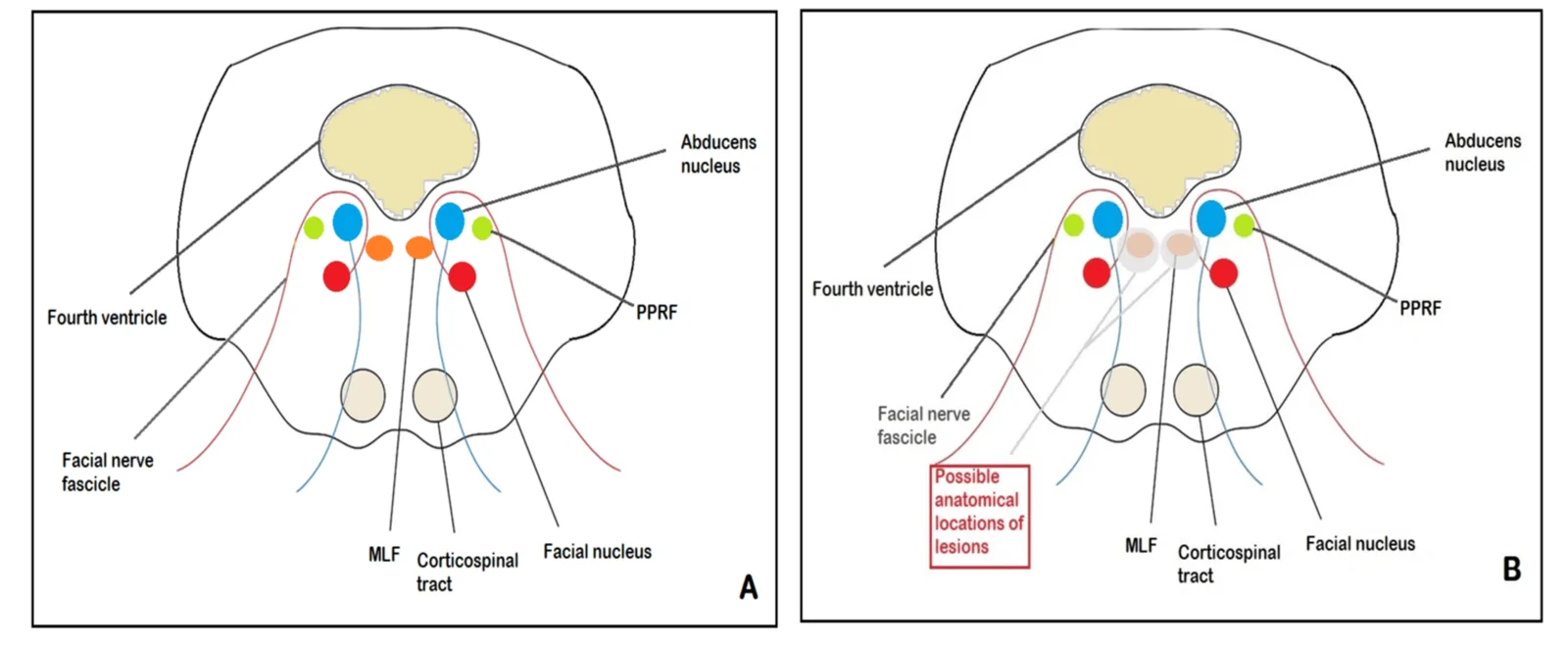
Schematic axial diagrams of the pontine tegmentum at the level of the facial colliculus. (A) Normal pontine tegmental anatomy. (B) Possible anatomical location of lesions responsible for WEBINO syndrome with facial diplegia in our case, involving the bilateral medial longitudinal fascicles (MLFs) and facial nerve fascicles. WEBINO: Wall-eyed bilateral internuclear ophthalmoplegia; PPRF: paramedian pontine reticular formation. The figure was created using Microsoft Paint.

Brain magnetic resonance imaging (MRI) revealed two bilateral acute ischemic infarcts in the dorsal pontine tegmentum, characterized by hyperintensity in the diffusion-weighted-imaging (DWI) sequence with corresponding decreased apparent diffusion coefficient (ADC). Magnetic resonance angiography (MRA) demonstrated intracranial atherosclerosis in the proximal third of the basilar artery and the right vertebral artery. A chronic left thalamic lacunar infarct was also identified. Susceptibility-weighted imaging demonstrated multiple bilateral deep cerebral microbleeds consistent with small vessel disease (Figure 3). A schematic representation of the pontine tegmental anatomy and the proposed anatomical location of the lesions are shown in Figure 4.

Electrocardiogram (ECG) showed sinus rhythm. Transthoracic echocardiography revealed no abnormalities. Routine laboratory investigations showed no abnormalities apart from an elevated HbA1c consistent with poorly controlled diabetes. The results are summarized in Table 1.

**Table 1:** Laboratory parameters. HbA1c: hemoglobin A1C; LDL: low-density lipoprotein; HDL: high-density lipoprotein.

Parameter	Patient values	Reference range
Fasting blood glucose	0.95 g/L	<1.26 g/L
HbA1c	8.5%	4-6%
Total cholesterol	1.44 g/L	1.4–2 g/L
LDL-cholesterol	0.80 g/L	<1.60 g/L
HDL-cholesterol	0.42 g/L	0.4–0.6 g/L
Triglyceride	1.11 g/L	0.5–1.5 g/L

Given the clinical presentation and imaging findings, an acute dorsal pontine tegmental infarction was diagnosed, presenting as WEBINO syndrome combined with facial diplegia. The two DWI/ADC-positive foci were presumed to correspond to the territories of two distinct paramedian perforating arteries.

Due to the 24-hour delay from symptom onset, she was outside the standard window for intravenous thrombolysis. No large vessel occlusion was identified on vascular imaging; therefore, mechanical thrombectomy was not indicated.

Dual antiplatelet therapy with acetylsalicylic acid (160 mg once daily) and clopidogrel (75 mg once daily) was initiated for three months, with strict control of cerebrovascular risk factors and rehabilitation therapy. This therapeutic decision was based on the significantly higher risk of early ischemic recurrence associated with symptomatic intracranial atherosclerosis compared with other stroke mechanisms, as reported in several studies [8], and the lower hemorrhagic risk with the deep distribution of cerebral microbleeds observed in our patient, consistent with hypertensive small-vessel disease, compared with the lobar pattern typically associated with cerebral amyloid angiopathy [9].

At four months of follow-up, her condition remained stable, with an mRS score of 4, attributable to her residual right hemiparesis and persistent diplopia.

## Discussion

The pontine tegmentum contains structures involved in the control of horizontal conjugate eye movements, including the MLF, paramedian pontine reticular formation (PPRF), and abducens nucleus.

The MLF is a paired, heavily myelinated tract located in the paramedian dorsal tegmentum of the midbrain and pons, ventral to the floor of the fourth ventricle. It coordinates horizontal conjugate eye movements by interconnecting the PPRF and abducens nucleus complex with the contralateral oculomotor nucleus [1,2]. The abducens nucleus, located at the floor of the fourth ventricle in the lower pons, contains two neuronal populations: abducens motoneurons, whose axons form the abducens nerve and innervate the ipsilateral lateral rectus muscle, and internuclear neurons, whose axons decussate and ascend in the contralateral MLF to the oculomotor nucleus, which innervates the contralateral medial rectus muscle [10]. The PPRF, the supranuclear horizontal gaze center, receives input from the frontal and parietal eye fields and projects to the ipsilateral abducens nucleus, producing simultaneous ipsilateral eye abduction and contralateral eye adduction via the MLF, thereby generating horizontal conjugate gaze [2]. The facial motor nucleus is also located in the pontine tegmentum. Its fibers encircle the abducens nucleus, forming the internal genu of the facial nerve and contributing to the facial colliculus [3].

Because of the close anatomical proximity of these structures, lesions involving the pontine tegmentum can produce a variety of clinical manifestations depending on the structures affected. A unilateral MLF lesion causes internuclear ophthalmoplegia (INO), characterized by limited adduction of the ipsilateral eye and abducting nystagmus of the contralateral eye during horizontal gaze [1]. Bilateral MLF involvement results in WEBINO syndrome, characterized by bilateral adduction deficits, bilateral abducting nystagmus, and exotropia in primary gaze, with preserved abduction in both eyes [11]. A lesion extending beyond the MLF and involving the facial nerve nucleus or fascicles can consequently produce facial weakness; when bilateral, this can result in facial diplegia.

WEBINO syndrome associated with facial diplegia represents an extremely rare entity. To our knowledge, three previously reported cases were identified through a literature search of PubMed/MEDLINE, from database inception in July 2026. The clinical, radiological, and outcome characteristics of these three cases are summarized in Table 2.

**Table 2:** Summary of previously reported cases of WEBINO syndrome with facial diplegia. WEBINO: Wall-eyed bilateral internuclear ophthalmoplegia; INO: internuclear ophthalmoplegia

Case	Author, year	Age/sex	Medical history	Clinical presentation	MRI findings	Etiology	Outcome
1	Kamogawa et al. [4], 2009	50/M	Secondary progressive multiple sclerosis	Progressive WEBINO with facial diplegia, pseudobulbar palsy, and spastic tetraparesis	Paramedian pontine tegmental T2-hyperintense lesion without enhancement; disseminated cerebral white matter and cervical spinal cord lesions	No acute demyelinating lesion clearly identified	Mild improvement in bilateral adduction and tetraparesis after steroid pulse therapy; WEBINO persisted
2	Im et al., [5], 2020	62/F	Hypertension, type 2 diabetes mellitus	Sudden-onset WEBINO and gait disturbance, followed by facial diplegia on day 3	Midline dorsal pontine infarction; posterior circulation stenosis	Ischemic stroke; possible perforating artery variant supplying both sides of the dorsal tegmentum	Aspirin, clopidogrel, and lipid-lowering therapy; gradual recovery after two weeks
3	Dominguez et al., [6], 2023	66/M	Not reported	Sudden-onset instability and WEBINO, followed by facial diplegia and dysarthria seven days later	Lacunar infarction in the lower paramedian pons; subsequent MRI showed no increase in infarct volume	Ischemic stroke; possibly cytotoxic mechanism	Partial recovery; at three months, facial diplegia improved, but right INO and mild right lower facial paresis persisted

The previously reported cases had different presumed etiologies: ischemic pontine lesion in the cases of Im et al. and Dominguez et al. [5,6] and secondary progressive multiple sclerosis with a possible acute demyelinating lesion in the case reported by Kamogawa et al. [4]. In the two ischemic cases [5,6], the facial diplegia developed after the onset of WEBINO syndrome, on day 3 and day 7, respectively. In the case reported by Im et al. [5], follow-up DWI revealed extension of the initial pontine lesion. In contrast, according to Dominguez et al. [6], progressive symptoms occurred without radiological infarct extension, as follow-up neuroimaging showed no increase in infarct volume. The authors hypothesized that this progression might reflect cytotoxic edema associated with the ischemic lesion. The timing of WEBINO and facial diplegia onset was not specified in the case reported by Kamogawa et al. [4].

In the previously reported cases [4-6], MRI demonstrated a single pontine lesion involving the dorsal pontine tegmentum. Paramedian pontine perforating arteries usually arise as paired, separate vessels, each supplying its respective side [12]. Consequently, paramedian pontine infarctions are predominantly unilateral. Im et al. [5] proposed that an anatomical variation in the pontine perforating arteries, whereby a single paramedian perforating artery supplies both sides of the dorsal tegmentum, could explain the bilateral clinical manifestations despite the presence of a single midline lesion on MRI.

In our case, WEBINO syndrome and facial diplegia occurred simultaneously. Brain MRI revealed two distinct bilateral ischemic lesions within the dorsal pontine tegmentum with evidence of posterior circulation atherosclerosis. We hypothesize that an anatomical variant in which the pontine perforating arteries share a common origin from the basilar artery could explain the bilateral ischemic lesions observed on MRI, conceptually analogous to the artery of Percheron in the thalamic circulation. However, this hypothesis requires angiographic or anatomical confirmation.

Other pontine tegmental syndromes have also been described. The “one-and-a-half syndrome,” first described by Fisher in 1967 [13], remains a reference syndrome within the spectrum of pontine tegmental syndromes. It results from a lesion involving the PPRF or abducens nucleus together with the ipsilateral MLF; it is characterized by ipsilateral horizontal gaze palsy associated with ipsilateral INO, with only contralateral eye abduction preserved (1+1/2 = 1.5). The association of one-and-a-half syndrome with ipsilateral peripheral facial palsy is termed the “eight-and-a-half syndrome” (1/2+7 = 8.5), first described by Eggenberger in 1998 [14]. The “fifteen-and-a-half syndrome” (1+ 1/2+7+7 = 15.5) reported by Bae et al. [15] combines the one-and-a-half syndrome with facial diplegia. The “sixteen syndrome” (1/2+1/2+1/2+1/2+7+7=16) reported by Connors et al. [16] is characterized by bilateral horizontal gaze impairment associated with facial diplegia due to a lesion of the posterior pontine tegmentum.

## Conclusions

WEBINO syndrome with concomitant facial diplegia constitutes a rare and potentially underrecognized manifestation of a pontine tegmental lesion. Our case presents a distinct imaging pattern and raises the possibility of an anatomical variant involving the vascular supply of the paramedian pontine tegmentum.
